# Highly Durable Additively Manufactured Membrane-Free Electrolyzer

**DOI:** 10.1021/acsomega.5c01163

**Published:** 2025-05-31

**Authors:** Matthew J. Whittingham, Eric M. Brack, James Waggett, Robert D. Crapnell, Craig E. Banks, Samuel J. Rowley-Neale

**Affiliations:** † Faculty of Science and Engineering, 5289 Manchester Metropolitan University, Dalton Building, Chester Street, Manchester M1 5GD, Great Britian; ‡ U.S. Army Combat Capabilities Development Command (DEVCOM)—Soldier Center, 10 General Greene Avenue, Natick, Massachusetts 01760, United States

## Abstract

We present a membrane-free water electrolyzer device that is wholly composed of additively manufactured components. Importantly, the anode and cathode are additively manufactured using filaments of Inconel-625 (I-AME) via fused filament fabrication. The I-AMEs exhibit efficient electrocatalysis toward major reactions within water electrolyzer devices, namely, the hydrogen evolution reaction on the cathode and the oxygen evolution reaction on the anode, in both acidic and alkaline electrolytes. The I-AMEs exhibit excellent stability with no decline in their electrochemical signal output during an 80 h chronoamperometry procedure and 192 h of submersion in 0.5 M H_2_SO_4_. Key insights into the effect of electrode design and architecture within flow devices are presented with computational fluid dynamic modeling revealing optimal electrode designs to maximize electrode–electrolyte interaction. The additively manufactured electrolyzer device was shown to be capable of producing 100.8 mL/h and 36 mL/h of hydrogen and oxygen, respectively, at a low current density of ca. 5 mA cm^–2^. The herein described additive manufactured water electrolyzer device has the potential to produce significant quantities of hydrogen and oxygen gas in remote scenarios without the requirement of complex and costly technologies.

## Graphical abstract

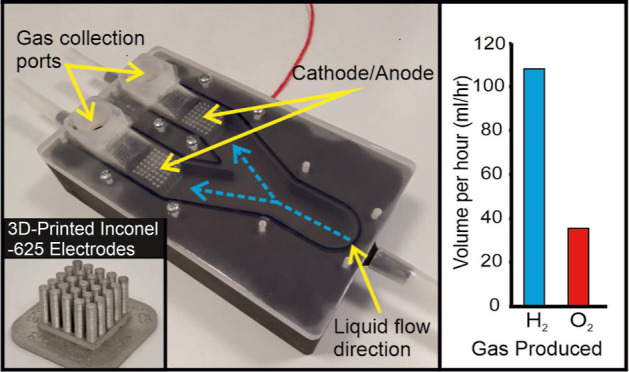

## 1 Introduction

Currently, the major obstacle to the ubiquitous implementation of renewable energy sources, such as solar, wind, wave, etc., is that their production is often ill correlated to consumer demand.^1,2^ Utilizing the energy generated by such renewables to create hydrogen, via water electrolysis, is a promising method of storing the generated power within a versatile energy vector for later use as and when required.^3^ The two most commercially viable electrolyzer technologies are proton exchange membrane (PEM) electrolyzers^4,5^ and alkaline water electrolysis (AWS)^6,7^ of which anion exchange membrane (AEM) electrolyzers^8^ represent a promising technology. In both electrolyzer technologies, hydrogen is generated at the cathode via the hydrogen evolution reaction (HER) and oxygen is generated at the anode via the oxygen evolution reaction (OER).^9–11^ These reactions are typically catalyzed by precious noble metal catalysts,^12,13^ which, when combined with the complexity of design and production of electrolyzer cells, result in the production costs of green hydrogen being significantly more than the fossil fuel (FF)-based counterparts.^2^ Given this, only 4% of the global production of hydrogen is via water electrolysis.^14,15^ In order to make green hydrogen an economically viable alternative to FFs, research efforts have focused on finding cheaper, more abundant catalytic alternatives to the precious metals currently utilized as well as working to refine and simplify the electrolyzer design and manufacturing process.

There is a plethora of potential nonprecious metal (NPM) catalytic replacements for the anodic and cathodic material in both PEM and AWS electrolysis systems reported within the literature.^16–18^ For example, transition-metal dichalcogenides (TMD),^19–21^ transition-metal phosphides (TMP),^22,23^ and metal–organic framework derivatives (MOF)^24,25^ have all been utilized to produce catalysts that exhibit Pt-like activity toward the HER within PEM electrolyzers. However, in the majority of studies, for both PEM and AWS NPM electrode catalysts, they exhibit initially promising electrocatalytic properties but fail to reach or do not disclose the catalysts stability testing regime necessary for implementation within their respective commercial electrolyzer applications.^16^ This significantly limits the potential for their application beyond the laboratory. Consideration therefore must be given to the intended application of the electrolyzer device for a compromise between cost, availability, activity, and stability of the utilized materials to be made. This work aims to produce an electrolyzer capable of being manufactured and deployed within the field, for use in disaster relief scenarios, such as refugee camps, where the quantity of hydrogen produced is sufficient for the daily needs (i.e., cooking, heating, etc.) for an individual/household. Given this, the electrode materials must be low cost, moderately catalytic, and highly stable in both acidic and alkaline electrolyzer devices. A material that can potentially offer these properties is “Inconel 625”, which is a nickel (Ni)-based superalloy that has been strengthened typically through the addition of 20–23 wt % chromium, 8–10 wt % molybdenum, and 3.14–4.15 wt % niobium.^26,27^ Inconel 625 is known for its exceptional corrosion resistance, high mechanical strength, and excellent thermal stability, making it a widely used material in extreme environments. Its high nickel and chromium contents provide resistance to oxidation and corrosion, even in highly aggressive conditions, including acidic and saline environments. Additionally, the presence of molybdenum and niobium enhances its strength through solid solution strengthening, allowing it to maintain its structural integrity at elevated temperatures and under high-stress conditions. Due to these properties, Inconel 625 has been explored for various electrochemical applications, including water electrolysis, where durability and resistance to degradation are critical. However, its application in water electrolysis remains limited due to factors such as its relatively high cost and potential surface passivation, which can reduce the electrocatalytic activity over time. To enhance its performance through surface modifications or alloying strategies, further studies are needed to optimize its electrochemical behavior and cost-effectiveness for large-scale hydrogen production.^26,27^ As a result of the materials desirable properties, it has been utilized within a plethora of industries such as aerospace,^28^ nuclear,^29^ and petrochemical.^30^ Inconel 625 has seen limited application within electrolyzers that generate hydrogen at high pressures (>59.2 MPa hydrogen gas at R.T using Sieverts law) due to hydrogen embrittlement inducing grain boundary fractures.^31^ There are however several studies within the literature where it has been effectively employed as an electrode material for a water splitting device, such as by Allebrod et al.,^32^ who demonstrated that an alkaline electrolysis cell comprising an Inconel 625 foam cathode and nickel foam anode displayed high electrical efficiency, with the Inconel cathode exhibiting an HER current density of 100 mA cm^–2^ at an overpotential of −40 mV and a Tafel slope of 91 mV dec^–1^.

The use of additive manufacturing offers several advantages over traditional manufacturing methods, making it an increasingly preferred choice in various industries. Unlike subtractive techniques, which involve cutting away material from a solid block, additive manufacturing builds components layer by layer, minimizing material waste and enabling complex geometries that would be difficult or impossible to achieve with conventional methods. This flexibility allows for rapid prototyping, customization, and on-demand production, reducing the lead times and costs associated with tooling. Additionally, additive manufacturing supports the use of advanced materials, such as polymers doped with carbon-based additives, to enhance mechanical, electrical, or thermal properties. Several studies have attempted to additively manufacture distinct components of an electrolyzer device, such as Chisholm et al.,^33^ who used additive manufacturing to fabricate prototype flow plates for use in a PEM electrolyzer, and Davis et al.,^34^ who utilized additive manufacturing to fabricate an electrolyzer chassis. Due to the architectural and manufacturing complexity of several components within an electrolyzer, such as the membrane electrode assembly (MEA) within a PEM electrolyzer (that consists of an ion exchange membrane, typically Nafion that is typically between 120 and 170 μm thick,^35^ with electrode layers hot-pressed on it), there have been very few attempts to additively manufacture complete electrolyzer devices. In order to overcome this issue, research has focused on reducing the architectural complexity of electrolyzers and removing the requirement for a membrane. Membraneless electrolyzers typically use flow-induced separation of the produced hydrogen and oxygen for downstream collection within separated collection chambers; this is possible due to the buoyancy of the gases within the electrolyte.^36,37^ The use of a membrane-free electrolyzer also eliminates the problem of generating hydrogen at high pressures and therefore avoids the potential degradation of the Inconel 625 electrodes due to embrittlement. Membrane-free electrolyzers present an alternative approach to conventional proton exchange membrane (PEM) and anion exchange membrane (AEM) electrolyzers, offering distinct advantages and challenges. Unlike PEM and AEM electrolyzers, which rely on ion-selective membranes to separate hydrogen and oxygen gases, membrane-free designs eliminate the need for costly and degradation-prone membranes, potentially reducing the system complexity and maintenance requirements. This design also allows for the use of a wider range of electrolytes and electrode materials, including those with higher stability under extreme conditions. Additionally, membrane-free electrolyzers can operate at higher current densities and enable more efficient gas bubble release, which may improve mass transport and overall efficiency. However, membrane-free systems also face significant challenges, particularly in gas separation and purity control. The absence of a physical membrane increases the risk of product gas crossover, which can lead to efficiency losses and safety concerns, especially at high operating pressures. Furthermore, achieving optimal electrode spacing and electrolyte flow dynamics is crucial to minimizing recombination losses and maintaining efficient ion transport. Compared to PEM and AEM electrolyzers, membrane-free designs are still in the early stages of development, with ongoing research focusing on improving gas separation techniques, optimizing electrolyte compositions, and enhancing long-term stability for practical deployment in large-scale hydrogen production. Bui et al.^38^ reported the additive manufacturing of a membrane-free electrolyzer that demonstrated an efficiency of 48% at 50 mA cm^–2^. While this study is elegant in its approach, the additively manufactured electrodes employed required a post-3D printing electrodeposition of nickel before they displayed adequate operational functionality. The inclusion of this post printing electrodeposition step detracts from the facile and low-cost nature of additive manufacturing, which reduces manufacturing costs. The reported stability of the electrodes, a 70 mV deviation over a 4 h testing period, was also not sufficient for implementation within commercial standard electrolyzers.

This paper reports the facile design and additive manufacturing, using FFF technology, of a membrane-free electrolyzer with Inconel 625 electrodes that allow for stable and efficient water splitting.

## 2 Results and Discussion

### 2.1 Physicochemical Characterization of the Inconel Filaments and Inconel-Additively Manufactured Electrodes

Initially, it was important to perform a physicochemical analysis of the Inconel filaments and the Inconel additively manufactured electrodes (I-AME), which was performed using SEM, EDX, and XPS. Figure 1A,B shows SEM images of an Inconel filament (I-F) and the I-AME, respectively.

**Figure 1:**
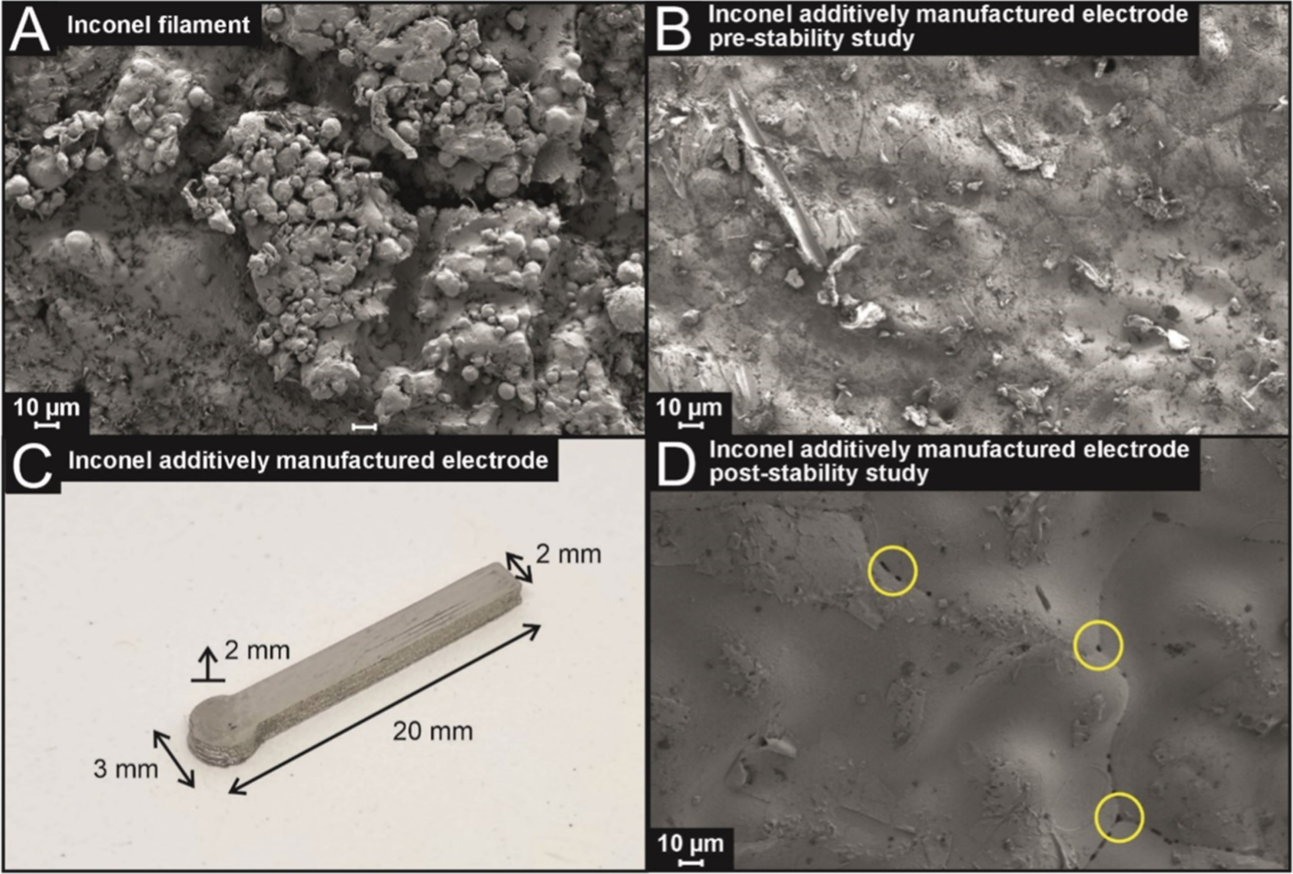
SEM images of the surfaces of the Inconel filament (A) and the Inconel additively manufactured electrode (I-AME) prestability testing (B). (C) An example I-AME utilized for the electrochemical studies. (D) An Inconel additively manufactured electrode (I-AME) post-stability testing (potential pitting at boundary sites highlighted in yellow).

The I-F has a rougher surface morphology when compared to the I-AME with round-edged “globule-like” structures evenly distributed across the surface that correspond to the binding polymer present within the filament. Post-additive manufacturing, the I-AME has a significantly smoother surface than the Inconel filament, which is likely due to the removal of the surrounding polymer globules by the sintering process. This is supported by EDX analysis (see Tables S1 and S2) of the I-F and I-AME, which shows a decrease in the presence of carbon that would be associated with the binding polymer and not the Inconel-625. XPS analysis was performed on an I-AME to assess its elemental composition, with the obtained XPS spectrum being shown in Figure S1. The elemental composition, while having a relatively high carbon content, which can be explained by incomplete polymer removal during the FFF additive manufacturing process, corresponds with that expected of Inconel-625.^39^ It is clear from the above analysis that the I-AME consists of Inconel-625 and that the subsequent electrochemical activity can be prescribed to this material.

### 2.2 Electrochemical Performance of the Inconel-Additively Manufactured Electrodes toward the HER and OER

The I-AMEs were characterized, with regard to their HER and OER activity. This was carried out using a typical three-electrode system, where a given I-AME (see Figure 1C) acted as the working electrode, with a large area carbon rod and a reversible hydrogen electrode (RHE) acting as the counter and reference electrodes, respectively. Note that all experiments were carried out in deoxygenated (nitrogen bubbled) 0.5 M H_2_SO_4_ and 1.0 M KOH, separately.

Figure 2A shows the obtained LSV for a polycrystalline Pt electrode and an I-AME between the potential range of +0.4 to −0.6 (vs RHE) in 0.5 M H_2_SO_4_. The HER activity of the Pt electrode was explored to benchmark the I-AME, and as expected, the Pt electrode displayed optimal HER activity with an onset potential and potential required to induce a current of −20 mA cm^–2^ of ca. −50 mV (vs. RHE) and ca. −127 mV (vs. RHE), respectively. In comparison, I-AME displayed slightly more electronegative values of ca. −184 mV (vs. RHE) and ca. −340 mV (vs. RHE), respectively. The Pt and I-AME reaction mechanism’s rate-limiting step was determined using Tafel analysis of the LSVs Faradaic regions; this yielded values of 29 and 76 mV dec^–1^, respectively, which suggest that Pt allowed the reaction to occur via the Volmer–Tafel discharge mechanism while the likely HER reaction pathway when using an I-AME is the Volmer–Heyrovsky discharge mechanism.^40^

**Figure 2:**
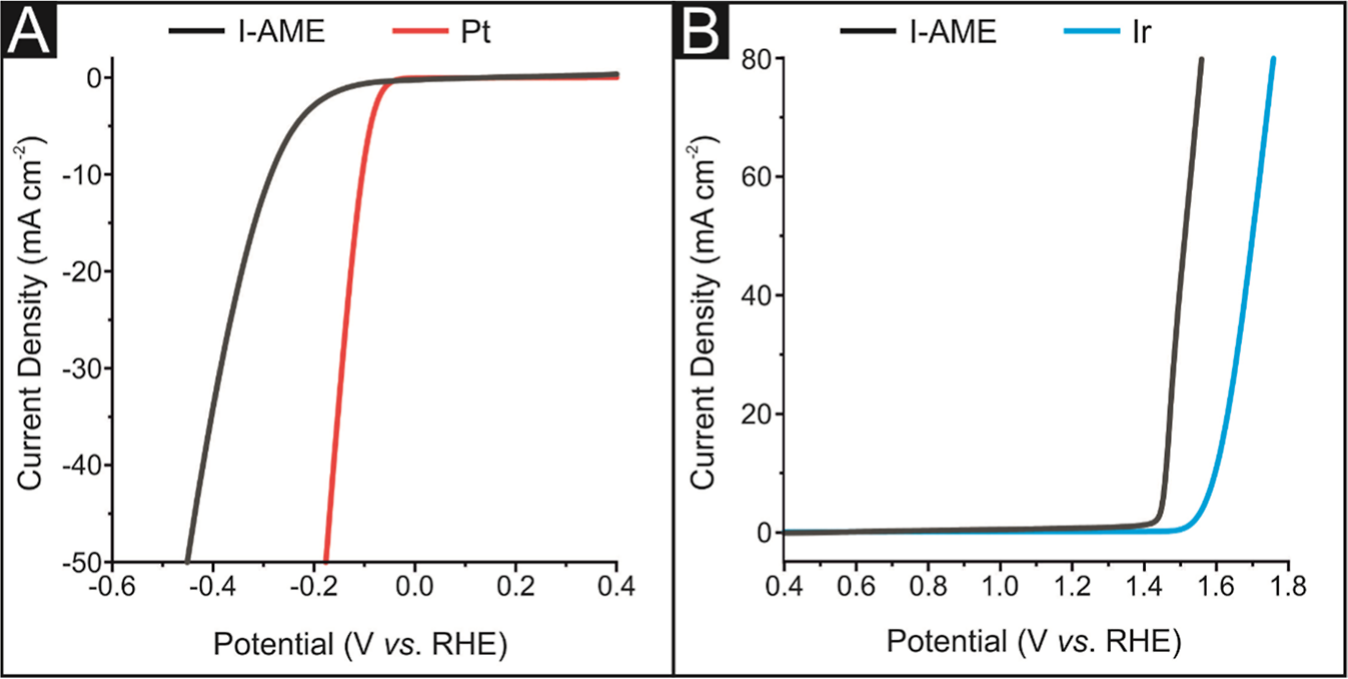
(A) Linear sweep voltammetry (LSV) exhibiting the onset potential of the HER between the potential range of 0.4 to −0.6 (vs RHE) using an I-AME (black line) and polycrystalline Pt electrode (red line), solution composition: 0.5 M H_2_SO_4_; scan rate: 25 mV s^–1^. (B) LSV exhibiting the onset potential of the OER between the potential range of 0.4 to 1.8 (vs RHE) using an I-AME (black line) and polycrystalline Ir electrode (blue line), solution composition: 0.5 M H_2_SO_4_; scan rate: 25 mV s^–1^.

Figure 2B shows the obtained LSV for a polycrystalline Ir electrode and an I-AME between the potential range of 0.4 to 1.8 (vs RHE) in 0.5 M H_2_SO_4_. The OER response of a polycrystalline Ir electrode was initially explored to allow for the I-AME activity to be benchmarked. The Ir electrode exhibited an OER onset potential and potential required to induce a current of +20 mA cm^–2^ of ca. + 1.56 V (vs. RHE) and ca. + 1.63 V (vs. RHE), respectively, while the I-AME displayed slightly less electropositive values of ca. 1.42 V (vs. RHE) and ca. 1.49 mV (vs. RHE), respectively. The OER reaction mechanism was determined via Tafel analysis of the LSV Faradaic regions; this yielded values of 65 and 106 mV dec^–1^ for the Ir electrode and I-AME, respectively, indicating that the rate-limiting step is O_2_ discharge for the I-AME while it is the initial H_2_O adsorption for Ir.^41^

Once the HER and OER activity of the I-AME had been deduced in an acidic electrolyte, it was determined whether the I-AME could also be applied within an alkaline electrolyte. To this end, Figure S2A shows the LSV response for a polycrystalline Pt electrode and an I-AME between the potential range of 0.4 to–0.6 (vs RHE) in 1.0 M KOH. The peak at ∼+1.4 V (vs RHE) is due to the electrochemical oxidation of Ni^2+^ to Ni^3+^, which has the oxidation of nickel species Ni^2+^(OH)_2_ to Ni^3+^OOH and Ni^4+^OO^–^, which results in the OER.^42^

The HER onset potential and potential required to induce a current of −20 mA cm^–2^ for the Pt electrode was observed to be ca. −65 mV (vs. RHE) and ca. −273 mV (vs. RHE), respectively, while the I-AME exhibited values that were more electronegative at ca. −428 mV (vs. RHE) and ca. −520 mV (vs. RHE), respectively. The Pt and I-AMEs reaction mechanism’s rate-limiting step was determined using Tafel analysis of the LSV Faradaic regions; this yielded values of 37 and 28 mV dec^–1^, respectively, which suggest that Pt allowed the reaction to occur via the Volmer–Heyrovsky mechanism, while the likely HER reaction pathway when using an I-AME is the Volmer–Tafel mechanism. Figure 4B shows the obtained LSV for a polycrystalline Ir electrode and an I-AME between the potential range of 0.8 to 1.8 (vs RHE) in 1.0 M KOH. The Ir electrode exhibited an OER onset potential and potential required to induce a current of +20 mA cm^–2^ of ca. 1.56 V (vs RHE) and ca. 1.63 V (vs RHE), respectively, while the I-AME displayed slightly less electropositive values of ca. 1.47 V (vs RHE) and ca. 1.52 mV (vs RHE), respectively. There is a clear oxidation peak at 1.4 (vs RHE) for the I-AME, which convolutes the OER onset. Future work is aimed at deconvoluting the source of this oxidation peak. The OER reaction mechanism was determined via Tafel analysis of the LSVs faradaic regions (see Figure 3B inset); this yielded values of 108 and 85 mV dec^–1^ for the Ir electrode and I-AME, respectively, suggesting that the initial H_2_O adsorption mechanism is the rate step for both Ir and I-AME.

**Figure 3:**
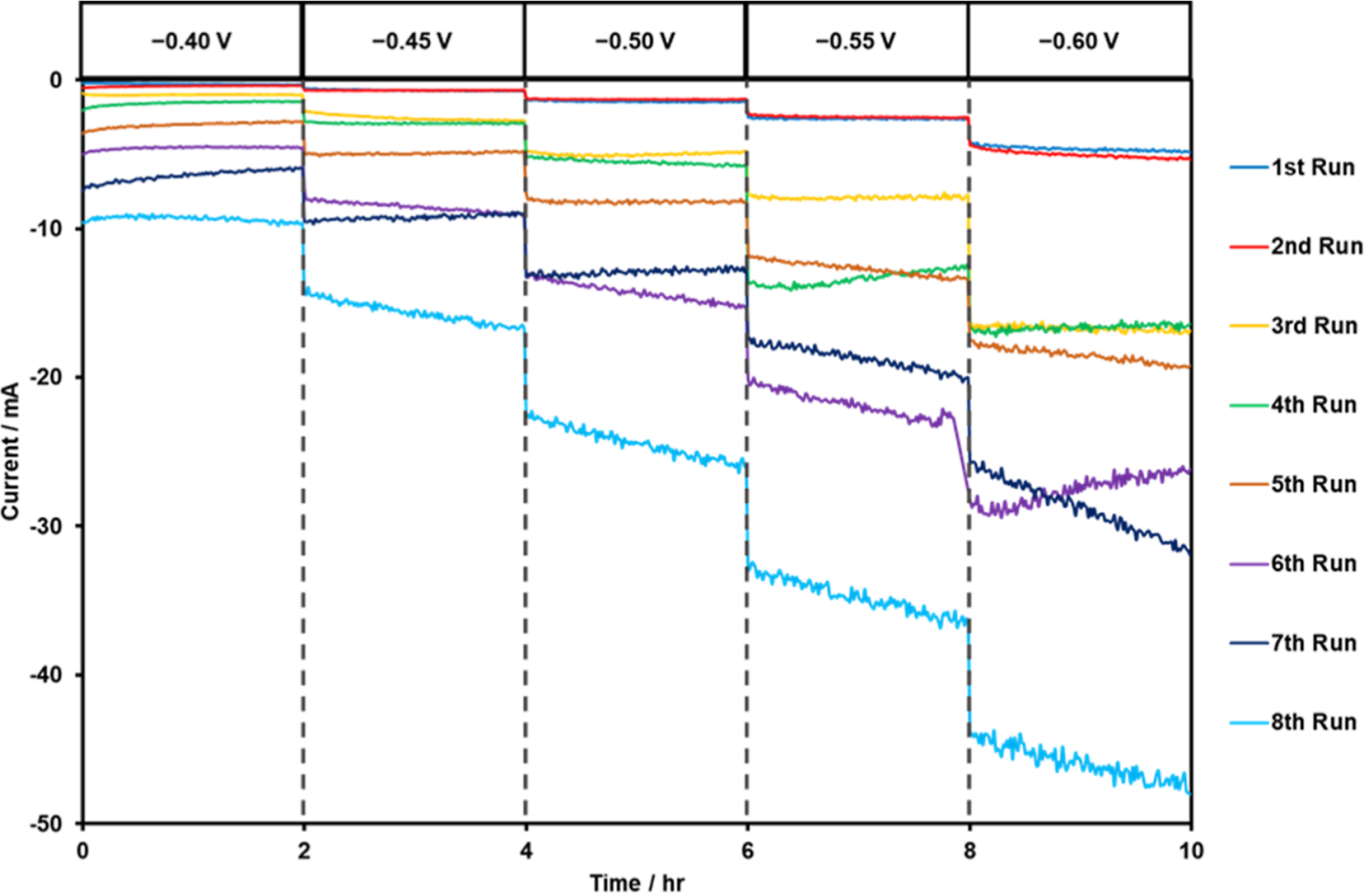
Chronoamperometry graph showing the performance of the I-additive manufactured electrode over 80 h in 0.5 M H_2_SO_4_. Voltage measured vs Ag/AgCl.

The results above demonstrate that when an I-AME is implemented as the working electrode toward the HER, the HER onset potential is reduced when an acidic, rather than alkaline, electrolyte is utilized (in this case by ca. 246 mV). Interestingly, the HER reaction mechanism favors an alkaline electrolyte with the reaction mechanism rate-limiting step being the desirable deadsorption Tafel step. This is supported by potential change required post-HER onset to reach an operational current density of 20 mA cm^–2^ to be significantly smaller when using an alkaline rather than acidic electrolyte at 290 and 90 mV, respectively.

From the above investigation, it is clear that the I-AMEs display efficient electrocatalysis toward the HER and OER in both acidic and alkaline electrolytes, displaying similar onset values to literature studies in which classical carbon-based electrodes were modified with well-known HER/OER electrocatalysts.^13,43^ Note that performing cyclic voltammetry and plotting log peak reduction height versus logscan rate revealed a linear response with a slope of ca. 0.5, which suggests the mass transport recorded from the I-AMEs was found to be diffusional in nature and that there was no trapped electrolyte/thin film effect occurring.

### 2.3 Electrochemical Stability of the Inconel-Additively Manufactured Electrodes

It was important to assess the stability of the I-AME electrochemical signal output. In order to do this, an I-AME was tested toward the HER within 0.5 M H_2_SO_4_ using chronoamperometry. As seen in Figure 3, the potential applied to the I-AME was increased stepwise from −0.4 V to −0.6 V (vs SCE) using 50 mV increments at 2 h intervals. This was repeated 8 times for a total of 80 h of stability testing. This experimental procedure was devised to mimic the partial/intermittent loads experienced by the electrolyzer within its infield application over the duration of a week. The obtained data can be viewed in Figure 3, and it is clear that the stepwise increase in potential resulted in an expected increase in the observed current in all instances. Note that the current is associated with the HER as the applied potentials are within the cathodic region post HER onset. There is a noticeable trend of increasing achievable current with sequential scans with the initial having a current of −5.35 mA at 10 h while the eighth scan has a current of −47.78 mA at 10 h.

In order to investigate why there was a significant increase in the achievable current, the estimated electroactive areas (*A*_real_) of the I-AME were determined pre- and post-stability testing using scan rate studies in the near ideal redox probe Ru­(NH_3_)_6_Cl_3_ and the quasi-reversible Randles–Ševčík equation.^44^ The obtained cyclic voltammogram scan rate studies can be viewed in Figure S3. Note that the I-AMEs both pre- and post-stability studies yielded a linear plot, with a slope less than 0.6, for log peak reduction current vs. log scan rate (see Figure S3B,D), revealing that in both cases the observed signal output is diffusional and that there is no observable thin film effect occurring. The determined values for the *A*_real_ for the I-AME pre- and post-stability were 0.17 cm^2^ and 0.37 cm^2^, respectively. We postulate that initially the I-AME structure may have the polymer binder present (associated with the filament), which dissolves in solution over the course of the stability testing; this leads to an increased number of exposed active sites, thus explaining the increase in achievable current. Figure 1D shows an SEM image of the I-AME post stability testing. A visual analysis of this image indicates that the surface of the I-AME appears to be smoother and with fewer surface morphological features and potential pitting at grain boundary sites; this potentially supports the above hypothesis. To further investigate this, XPS analysis was performed on an I-AME pre- and post-stability testing with the obtained spectrum shown in Figure 4. The carbon peak at ca. 285 eV is less prevalent in the post-stability spectrum, with the majority of the carbon present likely being from the filament additive; this supports our assertion that the polymer dissolved during the course of the stability study to reveal more Inconel active sites. Figure S4 shows the high-resolution XPS spectra for carbon on the I-AMEs pre- and post-stability testing.

**Figure 4:**
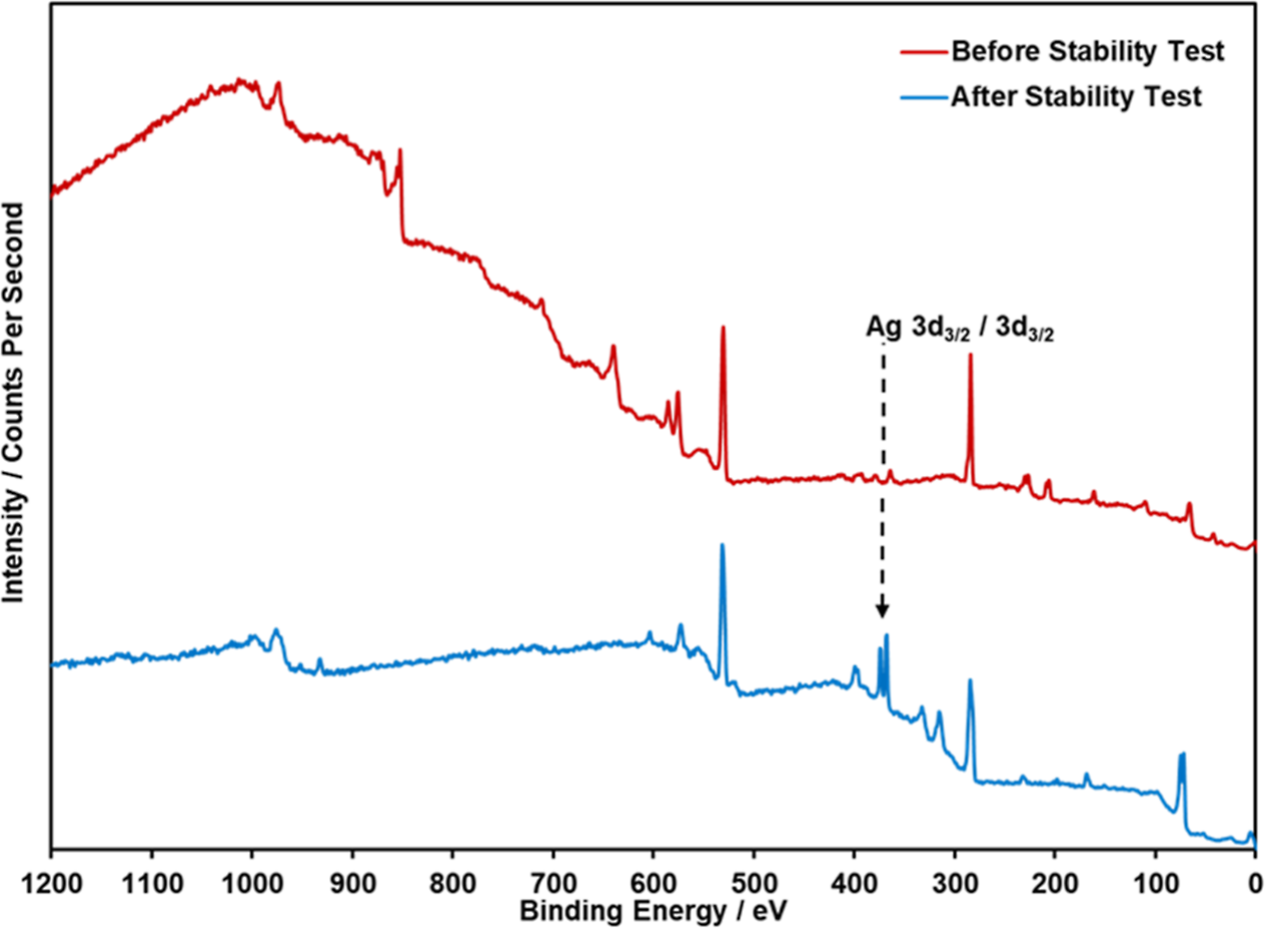
XPS survey scan comparison of the I-AME before (red) and after (blue) stability testing (adjusted intensity to allow comparison on the same graph).

This is further supported by EDX analysis that was carried out on the Inconel electrodes before and after electrochemical stability testing to determine the chemical composition of the electrode and any changes that occurred during experimentation. Compositions by weight percentage are listed in Table S2; note that the observed elemental composition closely matches the reference elemental composition data.^45^ Despite carbon generally being only a trace component (≤0.10 wt %) in Inconel 625, EDX confirmed a 14.07 wt % presence of carbon in the electrode before testing, which decreased by 3.01% in the I-AME post-stability study. It is proposed that there is residual polymer binder filament material left over from the I-AME production, and it represents a significant percentage of the electrodes weight. Interestingly, there is an Ag peak present in the post-stability study that is not present during the pre-stability; the presence of Ag on the I-AME surface is likely a result of leaching from the Ag/AgCl reference electrode of the order of 10^–6^ M based on the *K*_sp_, over the course of the 80 h stability study (Figure 4), which contributes to the generation of OER alongside nickel. In addition to the above, it would be of interest in future work to explore the stability of the I-AMEs within an alkaline medium.

### 2.4 Hydrogen Production Rate and Inconel-Additively Manufactured Electrodes CFD Design

Once the catalytic performance of the I-AMEs had been physiochemically and electrochemically characterized and shown to be electrocatalytically active toward the HER and OER. We have the opportunity to design and directly fabricate electrolyzer anodes and cathodes in any geometry/structure desired. Given this, Figures 5 and 6 demonstrate a proof-of-concept for a membraneless electrolyzer design that does not require any components that cannot be additively manufactured. Note that in this instance, to enable the electrodes to be visible through the lid of the electrolyzer, a clear resin lid was additively manufactured and then secured in place using screws. This is an unnecessary step but enabled gas production to be visibly observed. The I-AME electrolyzer was printed using selective laser sintering (SLS) and stereolithography (SLA) additive manufacturing using sintered PA12 nylon (for the main body) and clear resin (for the lid). The design (Figures 5 and 6) consists of a “Y-shaped” channel where electrolyte enters through the attached tubing (on the right), the flow splits at the fork, passes over the electrodes, and exits through the tubing (on the left) where electrolyte is cycled back around to the beginning. After the electrolyte passes over the electrodes gas will be produced, whereby the natural buoyancy will allow the gas to enter the collection tubes; in this case, liquid-filled syringes were utilized, from which the gas is isolated. Note that images of the electrolyzer device can be seen in Figure S5. The membraneless electrolyzer I-AME’s were manufactured as stated previously, while several distinct designs were explored via computational fluid dynamic (CFD) modeling to produce a design that would allow for a maximal electrode–electrolyte interaction. The modeling parameters can be found within the Supporting Information.

**Figure 5:**
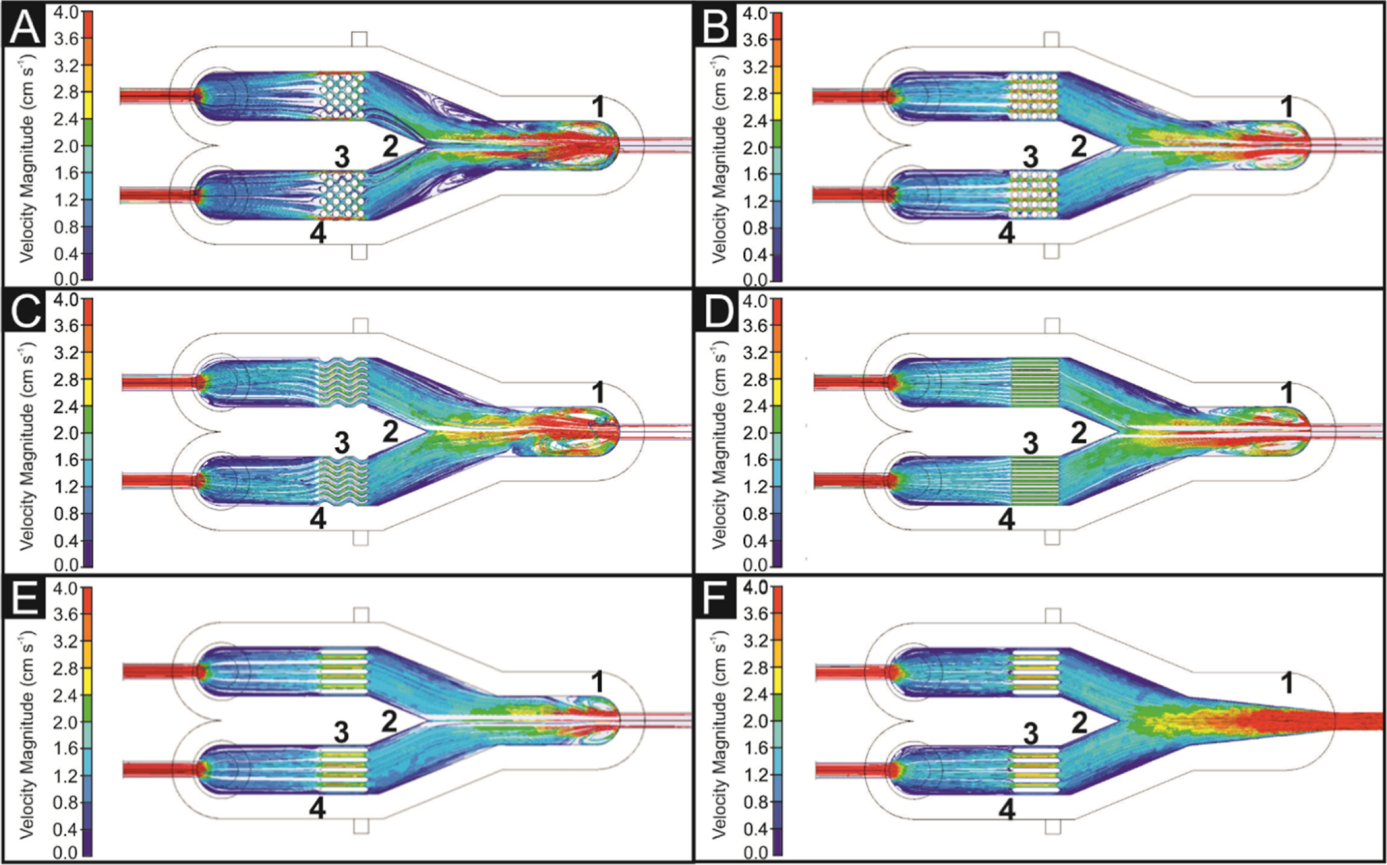
CFD simulations of Inconel additively manufactured electrode variations.

**Figure 6:**
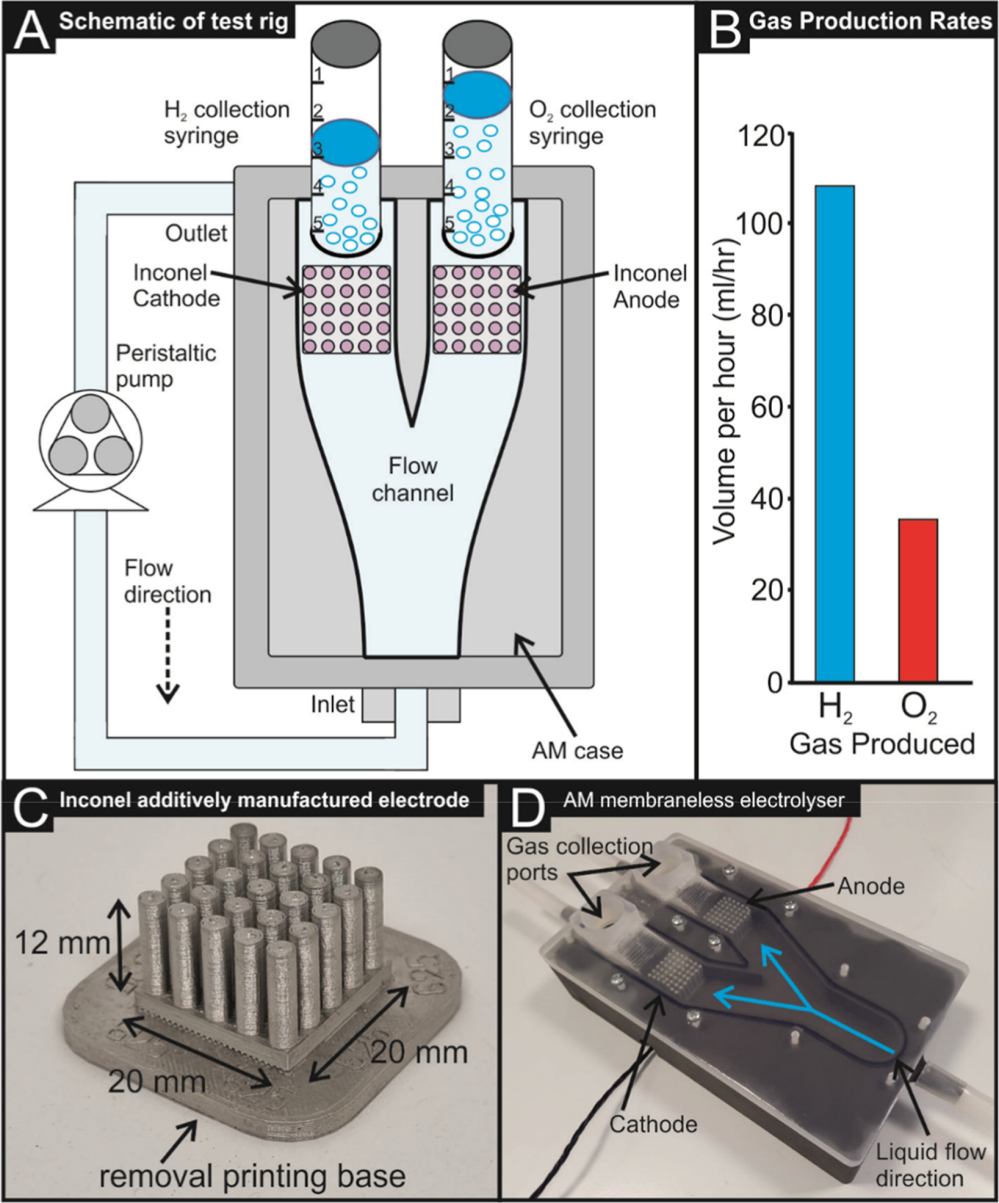
(A) Schematic of the test rig utilized to determine hydrogen production rates. (B) Hydrogen and oxygen production rates at a current density of 5 mA cm^–2^. (C) Image of I-AME utilized within the additively manufactured membraneless electrolyzer shown in (D).

Twenty-five 3 mm pin electrodes arranged in a staggered pattern are used in this cell design. The flow is turbulent after the inlet on the right side (see section 1–2). This turbulence continues more crucially over the flow separation (2)—this could potentially cause problems with gaseous products making their way back toward the inlets and mixing at the outlets. The overall darker blue color suggests that there is a large amount of slow (or stationary) fluid in the cell. Red sections around the outside edges of the electrodes suggest that the staggered pin design could be too restrictive to flow; fluid will take the path of least resistance, in this case around the electrodes (3). There are also notable dead spots on the trailing edges of the electrodes (4), which could prevent the electrode surfaces from being cleared of gases produced by fluid flow, leading to reduced efficiency from reduced reaction sites. The I-AME design in Figure 5B also uses 25 3 mm pin electrodes; however, the pins are arranged in a rectangular pattern rather than a staggered pattern. An overall lighter blue color and more laminar flow can be seen. The inlet flow (1) is still turbulent, however less so than with the staggered pin electrodes 4­(A); this slight inlet turbulence is simply a property of the expansion in cross-sectional area of the fluid from the tubing to the cell channel and is found in all further simulations. The flow rate remains higher from the inlet across the flow separation—shown by the comparatively greener coloration from section 1—2 versus (Figure 5A). A high interelectrode flow rate can be observed in section 3, showing that the rectangular pattern is far less restrictive than the staggered design; there is however dead spots between the pins and turbulence on the trailing edge of the electrodes. The electrode design pictured (5­(C)) features 0.8 mm “wavy” fins. 0.8 mm was designated as it would be 3D-printable with a dual-width extrusion from a standard 0.4 mm FFF nozzle. This design shows very even flow rate throughout the electrodes (section 3); The comparatively restrictive design also creates large amounts of turbulence at the inlet port (section 1). The flow over the fluid separator (section 2) is, however, both faster and more laminar than that in previous designs. This may indicate that inlet turbulence is a nonfactor in the design of membraneless hydrolyzers of this type. There are no noticeable dead spots on the electrodes (section 3), suggesting that this design of electrodes would provide a favorable refresh rate of reactant on the electrode surfaces and effectively move gaseous products from the surface to the outlets. There are two areas of slower flow on the interior sides of the cell gas traps/outlets (section 4). Figure 5D,E shows a simple straight fin electrode design. Figure 5D shows a fin width of 0.8 mm, allowing more fins per electrode, and Figure 5F shows a fin width of 2 mm. Both designs showcase very laminar flow throughout the majority of the hydrolyzer, but the 0.8 mm fins create higher turbulence at the inlet port (A) due to the increased resistance created by the denser fin spacing. The 2 mm fins (Figure 5F) show a much higher flow rate inside the electrode channels (B), which would lead to a faster refresh rate of the reaction sites compared to (Figure 5E); however, the 0.8 mm fins have a greater overall surface area. Testing these designs experimentally will show the output efficiency relationship between decreasing flow and increasing surface area, but it is clear that the fin designs are the superior designs in terms of flow rate and turbulence. Note that electrode design shown in Figure 5B was utilized for the studies below, but further studies are underway to optimize the I-AME, and the CFD modeling above highlights the importance of an electrode’s structure in its operational functionality.

The setup in Figure 6 was connected to a potentiostat, with the working (WE) and counter (CE). Chronoamperometry was performed on the cell, holding the cathode at −0.2 V (vs. RHE) for 150 s. Note this potential was chosen as it was the potential required for a current density of ca. 5 mA cm^–2^ to be achieved. Typically, a significantly higher current density would be desirable for such an experiment; however, ca. 5 mA cm^–2^ reduced the risk of over producing hydrogen within a lab-based scenario. Future work will seek to make a gas collection to enable high current densities to be explored. 1.5 mL of oxygen and 4.2 mL of hydrogen were produced after 150 s. This test was repeated 10×, using the same cathode and anode, with little to no significant variation in the gas production rates. Extrapolating these results, the production rates of the fully additively manufactured membraneless hydrolysis cell are 36 and 100.8 mL/h of oxygen and hydrogen, respectively, at 5 mA cm^–2^ current density. This gas production rate is comparable to other membraneless electrolyzer cells described within the literature.^37^

## 3 Conclusions

A proof of concept has been presented for an entirely additively manufactured membraneless water electrolyzer device that has the capacity to produce hydrogen and oxygen at a rate of 100.8 and 36 mL/h, respectively. The additively manufactured Inconel 625 electrodes implemented within this study were optimized with regard to their electrode–electrolyte interface design via the use computational fluid dynamic (CFD) modeling. The I-AMEs displayed no degradation in their electrochemical signal output over the course of 80 h chronoamperometry and 192 h of submersion in 0.5 M H_2_SO_4_; for practical use, stability will be explored further and will be reported in a future publication. In fact, an improvement in their electrochemical signal output was observed over the course of the stability study, which can likely be prescribed for dissolution of the remaining filament polymer within the I-AMEs and subsequent revealing of more Inconel electrolytically active sites. This was further supported by EDX that determined a decrease of 3.01% in the carbon present within the I-AMEs as well as an observed increase in the electrochemically active area from 0.17 cm^2^ to 0.37 cm^2^ for the I-AMEs pre- and post-stability study, respectively. Given this, the additively manufactured water electrolyzer device described herein has the potential to be developed and deployed within the field; for use in disaster relief scenarios, such as refugee camps, where the quantity of hydrogen produced is sufficient for daily needs (i.e., cooking, heating, etc.) of an individual/household. A cost-effective electrolyzer for disaster relief and refugee camps must prioritize affordable materials, energy efficiency, modularity, and ease of deployment. Future work could focus on integrating low-cost catalysts, optimizing power sources, and developing lightweight, scalable systems. Furthermore, in a membrane-free additive-manufactured electrolyzer, stability and performance are maintained through a combination of advanced design techniques that minimize ion migration and gas recombination. The electrodes are strategically spaced to ensure physical separation of hydrogen and oxygen gases, with optimized flow field designs that direct gas bubbles away from the reaction sites and a tight face plate. Electrolyte management also plays a key role, where controlled flow dynamics and pH buffering ensure distinct regions for cathode and anode reactions, preventing ion crossover. The design of the internal channels and the use of porous electrodes help manage gas evolution by guiding bubbles to separate collection zones, thus avoiding recombination. Additionally, catalysts and electrode materials are selected for their stability and efficiency, ensuring that only the desired reactions occur at each electrode. By integrating these strategies, the membrane-free electrolyzer achieves high efficiency and long-term stability while minimizing the potential for gas crossover and reducing the need for a physical membrane.

## 4 Experimental Section

### 4.1 Chemicals

All chemicals used were of analytical grade and were used as received from Sigma-Aldrich and used without any further purification. The filaments were purchased from Additive-X.^39^ All solutions were prepared with deionized water of resistivity not less than 18.2 MΩ cm^–1^ and were vigorously degassed prior to electrochemical measurements with high purity, oxygen-free nitrogen. All measurements were performed in 0.5 M H_2_SO_4_ or 1.0 M KOH. Note that the sulfuric acid solution and potassium hydroxide powder utilized were of the highest possible grade available from Sigma-Aldrich. Note that the HER and OER onset potentials denoted within the manuscript are defined as the potential at which the current initially deviates from the background current by a value of 25 μA cm^–2^, thus signifying the commencement of the Faradaic current associated with the HER and OER redox reactions.

### 4.2 Electrochemical Measurements

Electrochemical measurements were performed using an Autolab 100N potentiostat controlled by NOVA 2.1.7 (Utrecht, The Netherlands). All electrochemical measurements were performed using a typical three-electrode system in which a large area carbon electrode and a saturated calomel electrode (SCE) were utilized. Note that for the purpose of comparison, all potential (V) values were converted to ones representative of having used a reversible hydrogen electrode (RHE) reference. The I-AMEs were used as both the anode and cathode during the hydrolysis experiments being performed.

### 4.3 Physicochemical Characterization Equipment

Further details as to the specifications of Raman spectroscopy, scanning electron microscopy (SEM) with energy-dispersive X-ray microanalysis (EDX), and X-ray photoelectron spectroscopy (XPS) equipment used within this study to perform the physicochemical characterization are described within the Supporting Information.

## Supplementary Material



## Notes

Experimental section; CFD parameters; XPS survey spectrum of I-AME; linear sweep voltammetry; scan rate studies; fine-resolution XPS analysis for carbon on I-AME; and images of the electrolyzer device (PDF)

## References

[1] Arutyunov V. S., Lisichkin G. V. (2017). Energy resources of the 21st century: problems and
forecasts. Can renewable energy sources replace fossil fuels?. Russ. Chem. Rev..

[2] Hughes J. P., Clipsham J., Chavushoglu H., Rowley-Neale S. J., Banks C. E. (2021). Polymer electrolyte electrolysis: A review of the activity
and stability of non-precious metal hydrogen evolution reaction and
oxygen evolution reaction catalysts. Renewable
Sustainable Energy Rev..

[3] Oliveira A. M., Beswick R. R., Yan Y. (2021). A green hydrogen
economy for a renewable
energy society. Curr. Opin. Chem. Eng..

[4] Wang X. X., Swihart M. T., Wu G. (2019). Achievements, challenges
and perspectives
on cathode catalysts in proton exchange membrane fuel cells for transportation. Nat. Catal..

[5] Wan X., Liu X., Li Y., Yu R., Zheng L., Yan W., Wang H., Xu M., Shui J. (2019). Fe–N–C
electrocatalyst with dense active sites and efficient mass transport
for high-performance proton exchange membrane fuel cells. Nat. Catal..

[6] Liu X., Guo R., Ni K., Xia F., Niu C., Wen B., Meng J., Wu P., Wu J., Wu X. (2020). Reconstruction-Determined Alkaline Water Electrolysis at Industrial
Temperatures. Adv. Mater..

[7] Brauns J., Turek T. (2020). Alkaline Water Electrolysis Powered by Renewable Energy: A Review. Processes.

[8] Vincent I., Bessarabov D. (2018). Low cost hydrogen
production by anion exchange membrane
electrolysis: A review. Renewable Sustainable
Energy Rev..

[9] Zhang J., Zhao Y., Guo X., Chen C., Dong C.-L., Liu R.-S., Han C.-P., Li Y., Gogotsi Y., Wang G. (2018). Single platinum atoms immobilized
on an MXene as an efficient catalyst
for the hydrogen evolution reaction. Nat. Catal..

[10] Zhao S., Tan C., He C.-T., An P., Xie F., Jiang S., Zhu Y., Wu K.-H., Zhang B., Li H. (2020). Structural
transformation of highly active metal–organic framework electrocatalysts
during the oxygen evolution reaction. Nat. Energy.

[11] Chen P., Hu X. (2020). High-Efficiency Anion
Exchange Membrane Water Electrolysis Employing
Non-Noble Metal Catalysts. Adv. Energy Mater..

[12] Pfeifer V., Jones T. E., Wrabetz S., Massué C., Velasco Vélez J. J., Arrigo R., Scherzer M., Piccinin S., Hävecker M., Knop-Gericke A. (2016). Reactive oxygen species in iridium-based OER catalysts. Chem. Sci..

[13] Rowley-Neale S. J., Ratova M., Fugita L. T. N., Smith G. C., Gaffar A., Kulczyk-Malecka J., Kelly P. J., Banks C. E. (2018). Magnetron Sputter-Coated
Nanoparticle MoS2 Supported on Nanocarbon: A Highly Efficient Electrocatalyst
toward the Hydrogen Evolution Reaction. ACS
Omega.

[14] Chi J., Yu H. (2018). Water electrolysis
based on renewable energy for hydrogen production. Chin. J. Catal..

[15] Kumar S. S., Himabindu V. (2019). Hydrogen production by PEM water
electrolysis –
A review. Mater. Sci. Energy Technol..

[16] Hughes J. P., Clipsham J., Chavushoglu H., Rowley-Neale S. J., Banks C. E. (2021). Polymer electrolyte electrolysis:
A review of the activity
and stability of non-precious metal hydrogen evolution reaction and
oxygen evolution reaction catalysts. Renewable
Sustainable Energy Rev..

[17] Brauns J., Turek T. (2020). Alkaline Water Electrolysis
Powered by Renewable Energy: A Review. Processes.

[18] Zeng K., Zhang D. (2010). Recent progress in alkaline water electrolysis for hydrogen production
and applications. Prog. Energy Combust. Sci..

[19] Ahn B.-W., Kim T.-Y., Kim S.-H., Song Y.-I., Suh S.-J. (2018). Amorphous
MoS2 nanosheets grown on copper@nickel-phosphorous dendritic structures
for hydrogen evolution reaction. Appl. Surf.
Sci..

[20] Tsai C., Chan K., Abild-Pedersen F., Nørskov J. K. (2014). Active
edge sites in MoSe2 and WSe2 catalysts for the hydrogen evolution
reaction: a density functional study. Phys.
Chem. Chem. Phys..

[21] Wu Z., Fang B., Bonakdarpour A., Sun A., Wilkinson D. P., Wang D. (2012). WS2 nanosheets as a highly efficient electrocatalyst for hydrogen
evolution reaction. Appl. Catal., B.

[22] Schipper D. E., Zhao Z., Thirumalai H., Leitner A. P., Donaldson S. L., Kumar A., Qin F., Wang Z., Grabow L. C., Bao J. (2018). Effects
of Catalyst Phase on the Hydrogen Evolution
Reaction of Water Splitting: Preparation of Phase-Pure Films of FeP,
Fe2P, and Fe3P and Their Relative Catalytic Activities. Chem. Mater..

[23] Moon J.-S., Jang J.-H., Kim E.-G., Chung Y.-H., Yoo S. J., Lee Y.-K. (2015). The nature of active sites of Ni2P
electrocatalyst
for hydrogen evolution reaction. J. Catal..

[24] Zhu Z., Yang Y., Guan Y., Xue J., Cui L. (2016). Construction
of a cobalt-embedded nitrogen-doped carbon material with the desired
porosity derived from the confined growth of MOFs within graphene
aerogels as a superior catalyst towards HER and ORR. J. Mater. Chem. A.

[25] Fan L., Liu P. F., Yan X., Gu L., Yang Z. Z., Yang H. G., Qiu S., Yao X. (2016). Atomically isolated
nickel species anchored on graphitized carbon for efficient hydrogen
evolution electrocatalysis. Nat. Commun..

[26] Shankar V., Rao K. B. S., Mannan S. L. (2001). Microstructure and
mechanical properties
of Inconel 625 superalloy. J. Nucl. Mater..

[27] Guo Q., Li D., Guo S., Peng H., Hu J. (2011). The effect of deformation
temperature on the microstructure evolution of Inconel 625 superalloy. J. Nucl. Mater..

[28] Chintala A., Kumar K. M. T., Sathishkumar M., Arivazhagan N., Manikandan M. (2021). Technology Development for Producing
Inconel 625 in
Aerospace Application Using Wire Arc Additive Manufacturing Process. J. Mater. Eng. Perform..

[29] Rodriguez D., Merwin A., Karmiol Z., Chidambaram D. (2017). Surface chemistry
and corrosion behavior of Inconel 625 and 718 in subcritical, supercritical,
and ultrasupercritical water. Appl. Surf. Sci..

[30] Scrivani A., Ianelli S., Rossi A., Groppetti R., Casadei F., Rizzi G. (2001). A contribution to the
surface analysis
and characterisation of HVOF coatings for petrochemical application. Wear.

[31] Murakami, K. ; Yabe, N. ; Suzuki, H. ; Takai, K. ; Hagihara, Y. ; Wada, Y. Substitution of High-Pressure Charge by Electrolysis Charge and Hydrogen Environment Embrittlement Susceptibilities for Inconel 625 and SUS 316L. In Proceedings of the ASME 2006 Pressure Vessels and Piping/ICPVT-11 Conference; Volume 6: Materials and Fabrication; ASME, 2006, pp 563–570. 10.1115/pvp2006-icpvt-11-93397.

[32] Allebrod F., Chatzichristodoulou C., Mogensen M. B. (2013). Alkaline electrolysis cell at high
temperature and pressure of 250 °C and 42 bar. J. Power Sources.

[33] Chisholm G., Kitson P. J., Kirkaldy N. D., Bloor L. G., Cronin L. (2014). 3D printed
flow plates for the electrolysis of water: an economic and adaptable
approach to device manufacture. Energy Environ.
Sci..

[34] Davis J. T., Brown D. E., Pang X., Esposito D. V. (2019). High Speed Video
Investigation of Bubble Dynamics and Current Density Distributions
in Membraneless Electrolyzers. J. Electrochem.
Soc..

[35] Pantò F., Siracusano S., Briguglio N., Aricò A. S. (2020). Durability
of a recombination catalyst-based membrane-electrode assembly for
electrolysis operation at high current density. Appl. Energy.

[36] Lagadec M. F., Grimaud A. (2020). Water electrolysers
with closed and open electrochemical
systems. Nat. Mater..

[37] O’Neil G. D., Christian C. D., Brown D. E., Esposito D. V. (2016). Hydrogen Production
with a Simple and Scalable Membraneless Electrolyzer. J. Electrochem. Soc..

[38] Bui J. C., Davis J. T., Esposito D. V. (2020). 3D-Printed electrodes for membraneless
water electrolysis. Sustainable Energy Fuels.

[39] Additive-X. https://www.additive-x.com/shop/inconel-625-material.html (accessed Aug 25, 2023).

[40] Marković N., Grgur B., Ross P. N. (1997). Temperature-dependent hydrogen electrochemistry
on platinum low-index single-crystal surfaces in acid solutions. J. Phys. Chem. B.

[41] Hu J.-M., Zhang J.-Q., Cao C.-N. (2004). Oxygen evolution reaction on IrO2-based
DSA® type electrodes: kinetics analysis of Tafel lines and EIS. Int. J. Hydrogen Energy.

[42] Radinger H., Connor P., Tengeler S., Stark R. W., Jaegermann W., Kaiser B. (2021). Importance of Nickel
Oxide Lattice Defects for Efficient
Oxygen Evolution Reaction. Chem. Mater..

[43] Rowley-Neale S. J., Fearn J. M., Brownson D. A., Smith G. C., Ji X., Banks C. E. (2016). 2D molybdenum disulphide
(2D-MoS2) modified electrodes
explored towards the oxygen reduction reaction. Nanoscale.

[44] Ferrari A.
G.-M., Foster C. W., Kelly P. J., Brownson D. A. C., Banks C. E. (2018). Determination
of the Electrochemical Area of Screen-Printed Electrochemical Sensing
Platforms. Biosensors.

[45] Al
Ghafri S. Z. S., Munro S., Cardella U., Funke T., Notardonato W., Trusler J. P. M., Leachman J., Span R., Kamiya S., Pearce G. (2022). Hydrogen liquefaction:
a review of the fundamental physics, engineering practice and future
opportunities. Energy Environ. Sci..

