# Supplementary material for: Highly Durable Additively Manufactured Membrane-Free Electrolyzer
Source: ACS Omega. 2025 May 31;10(22):23089–98. doi: 10.1021/acsomega.5c01163 (PMC12163681; doi:10.1021/acsomega.5c01163)
Supplement: Supplementary file 1 [file ao5c01163_si_001.pdf]

Electronic supporting information for the following article:

Highly Durable Additively Manufactured Membrane Free Electrolyser

Matthew J. Whittingham,[1] Eric M. Brack,[2] James Waggett,[1] Robert D. Crapnell,[1] Craig E. Banks[1]* and Samuel J. Rowley-Neale[1]*

1: Faculty of Science and Engineering, Manchester Metropolitan University, Dalton Building, Chester Street, Manchester M1 5GD, Great Britian

2: U.S. Army Combat Capabilities Development Command (DEVCOM)─Soldier Center, 10 General Greene Avenue, Natick, Massachusetts 01760, United States

*To whom correspondence should be addressed.

Email: S.rowley-neale@mmu.ac.uk; Tel: ++(0)1612474622.

1. Experimental Section

1.1. Physicochemical characterization equipment

Scanning Electron Microscopy (SEM) micrographs were obtained using a Crossbeam 350 Focussed Ion Beam – Scanning Electron Microscope (FIB-SEM) (Carl Zeiss Ltd., Cambridge, UK) fitted with a field emission electron gun. Secondary electron imaging was completed using a Secondary Electron Secondary Ion (SESI) detector. Samples were mounted on the aluminium SEM pin stubs (12 mm diameter, Agar Scientific, Essex, UK) using adhesive carbon tabs (12 mm diameter, Agar Scientific, Essex, UK).

X-ray Photoelectron Spectroscopy (XPS) data were acquired using an AXIS Supra (Kratos, UK), equipped with a monochromatic Al X-ray source (1486.6 eV) operating at 225 W and a hemispherical sector analyser. It was operated in fixed transmission mode with a pass energy of 160 eV for survey scans and 20 eV for region scans with the collimator operating in slot mode for an analysis area of approximately 700 × 300 μm, the FWHM of the Ag 3d5/2 peak using a pass energy of 20 eV was 0.613 eV.

The energy efficiency is deduced from assuming theoretical voltage for water splitting is 1.23 V at 25°C which suggest that the additive manufacturing device is 65.7% efficient. This efficiency value is less than other membraneless electrolysers explored in the literature, such O'Neil et al.[1] The majority of such studies within the literature use more classical non additively manufactured electrode systems, whereas the electrolyser device described within this study utilise additively manufactured electrodes. The benefits of being able to fabricate the I-AMEs in-field is preferential to higher efficiency systems for specific use cases (*i.e.* immediate on-site energy production within disaster scenarios). Future work to improve the efficiency of the device is underway.

2. CFD Parameters

Several variations of membrane-less hydrolyser designs were designed using Autodesk Fusion360. For CFD purposes, the gas trap ports were blocked, as CFD cannot simulate mixed gases and fluids. It is assumed that the electrolyte does not interact with the gas stored in the gas traps – in experimental testing, the generated gases displace a syringe, and the fluid does not move past the gas traps, so this is an acceptable compromise for simulation. These models were then simulated using Autodesk CFD 2021 version 21.0, using moulded ABS as the solid material, and water as the fluid material. Inlet flow rate was set to 12 litres/hr – as used in prior experimental testing. Outlets were set to gauge pressure (0 Pa), and 500 cycles were run, with result converging after 200 iterations on average. Data was saved at 3s intervals.

Table S1. EDX analysis of the Inconel filament

Element	Atomic Mass %
C	33.36
O	14.97
Na	1.05
Al	2.89
Si	0.18
S	2.91
Cl	0.50
K	0.39
Cr	0.40
Fe	12.75
Ni	37.40
Nb	1.95

Table S2. EDX analysis of the I-AME pre and post stability study.

Element	I-AME wt.% (fresh)	I-AME wt.% (used)
Ni	53.81	54.45
Cr	18.24	19.25
C	14.07	11.06
Mo	7.33	7.73
Fe	2.58	2.66
Nb	2.19	2.49
Al	1.49	1.90
Si	0.33	0.47

Figure S1. XPS survey spectrum of an I-AME.

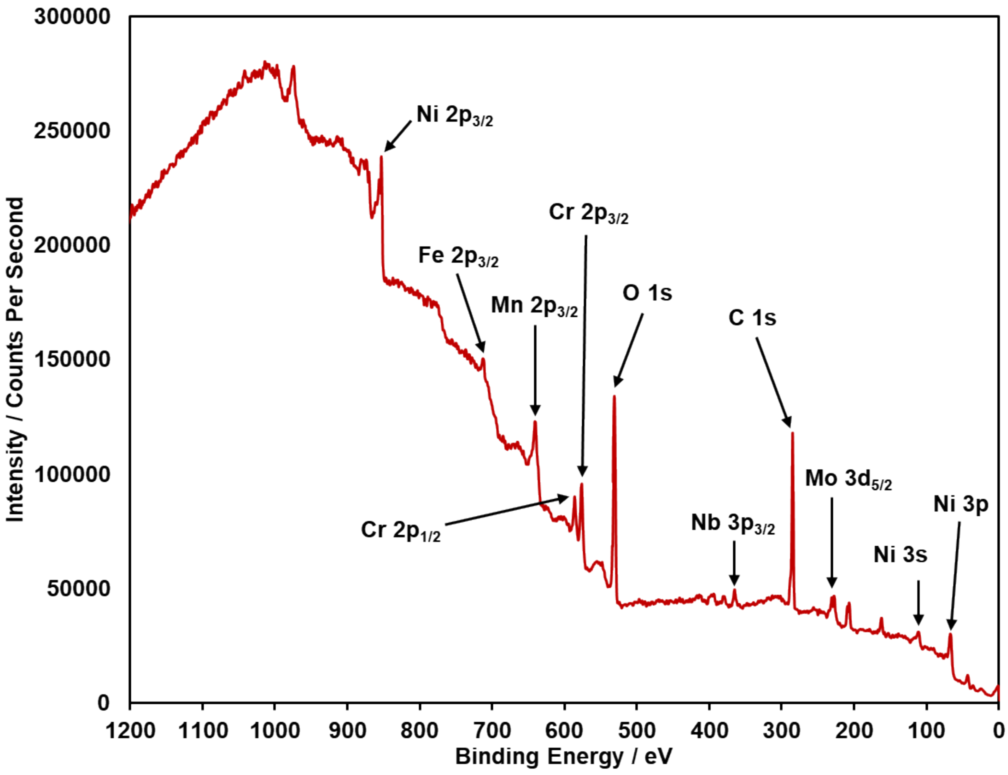

Figure S2. (A) Linear sweep Voltammetry (LSV) exhibiting the onset potential of the HER between the potential range of 0.4 to −0.6 (*vs.* RHE) using a I-AME (black line) and polycrystalline Pt electrode (red line), solution composition: 1.0 M KOH, scan rate: 25 mV s^{-1}. (B) LSV exhibiting the onset potential of the OER between the potential range of 0.8 to 1.8 (*vs.* RHE) using a I-AME (black line) and polycrystalline Ir electrode (blue line), solution composition: 1.0 M KOH, scan rate: 25 mV s^{-1}.

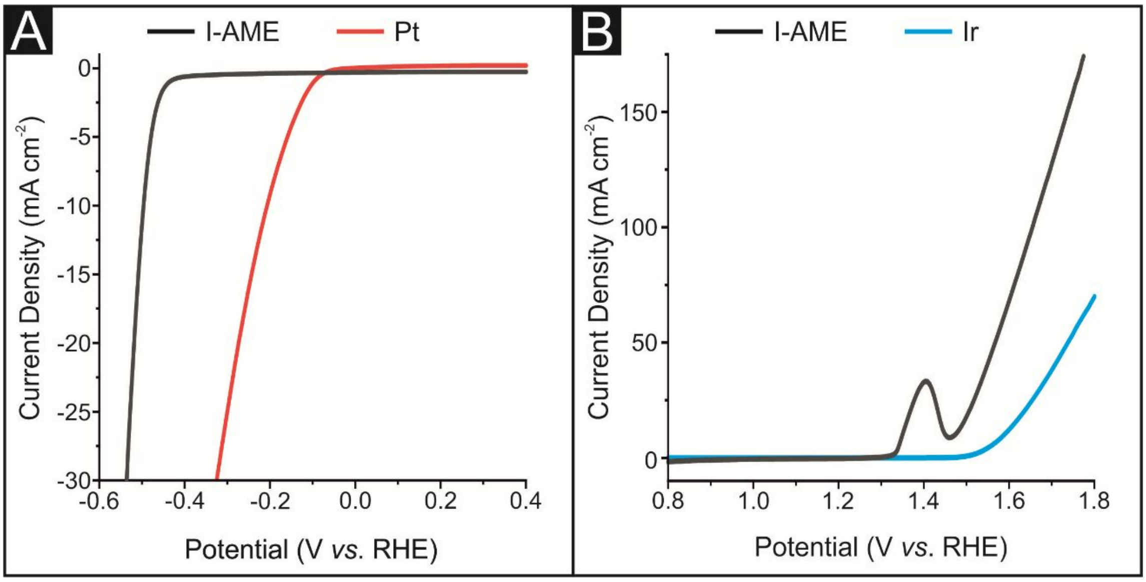

Figure S3. Scan rate studies using the redox probe 1mM $Ru(NH_3)_6Cl_3$ in 0.1 M KCl for (A) a I-AME pre-stability study and (B) post-stability study (C). (B) and (D) show the log of peak reduction currents observed in (A) and (B), respectively, against log scan rate utilised.

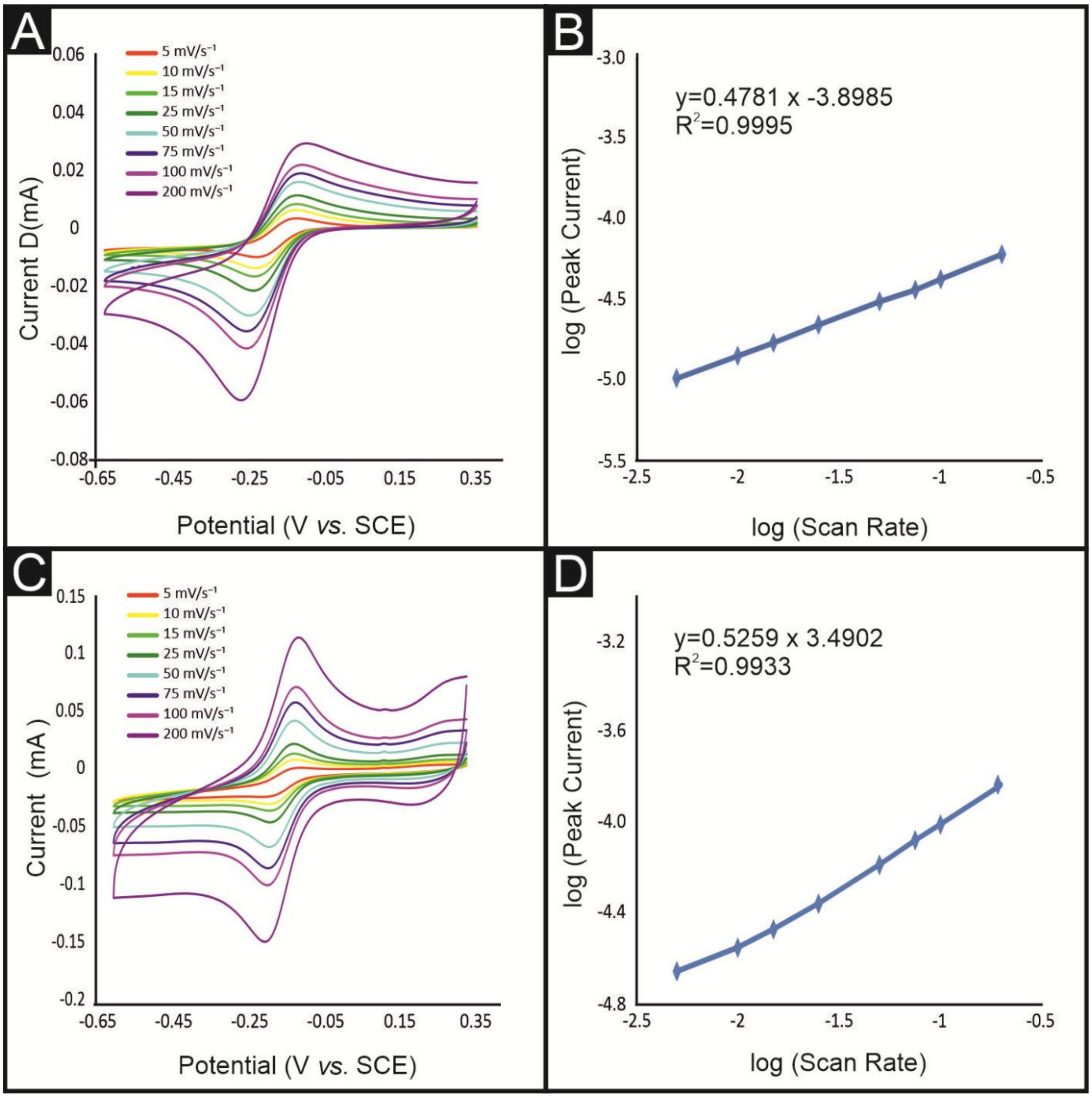

Figure S4. Fine resolution XPS analysis for carbon on an I-AME (A) pre and (B) post stability study.

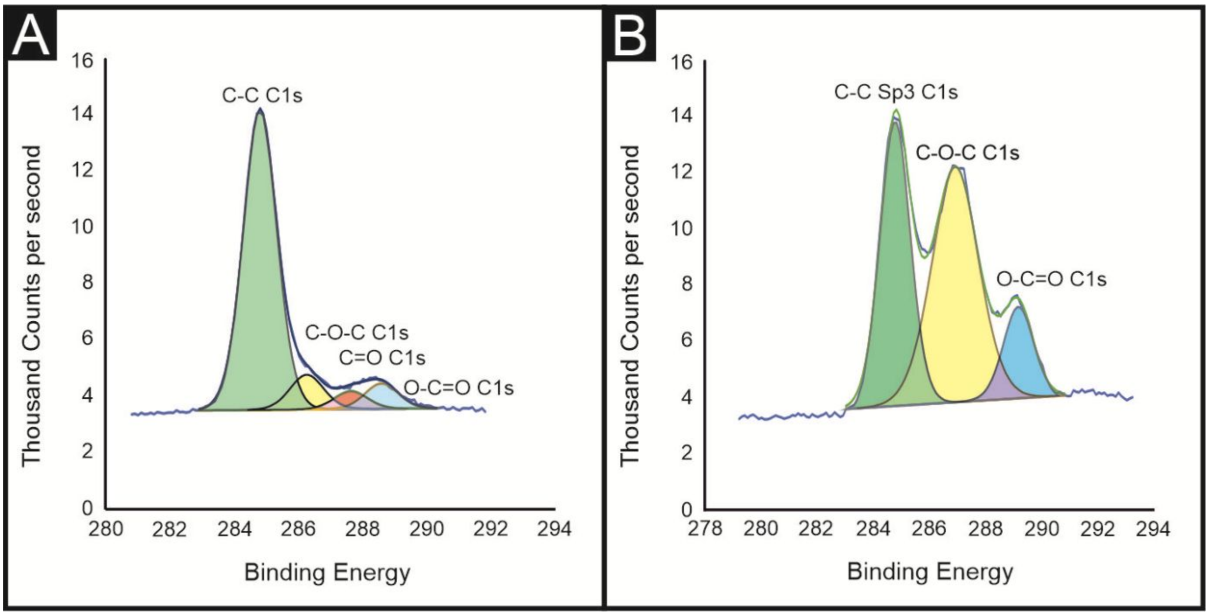

Figure S5. Images of the electrolyser device. (A) a CAD of the separate components of the electrolyser device, (B) CAD highlighting the electrical connections between the I-AMEs and potentiostat cabling, (C) photograph of the AM electrolyser device with the connected gas collection ports (liquid filled syringes), (D) photograph of the underside and electrolyser and the electrical connections.

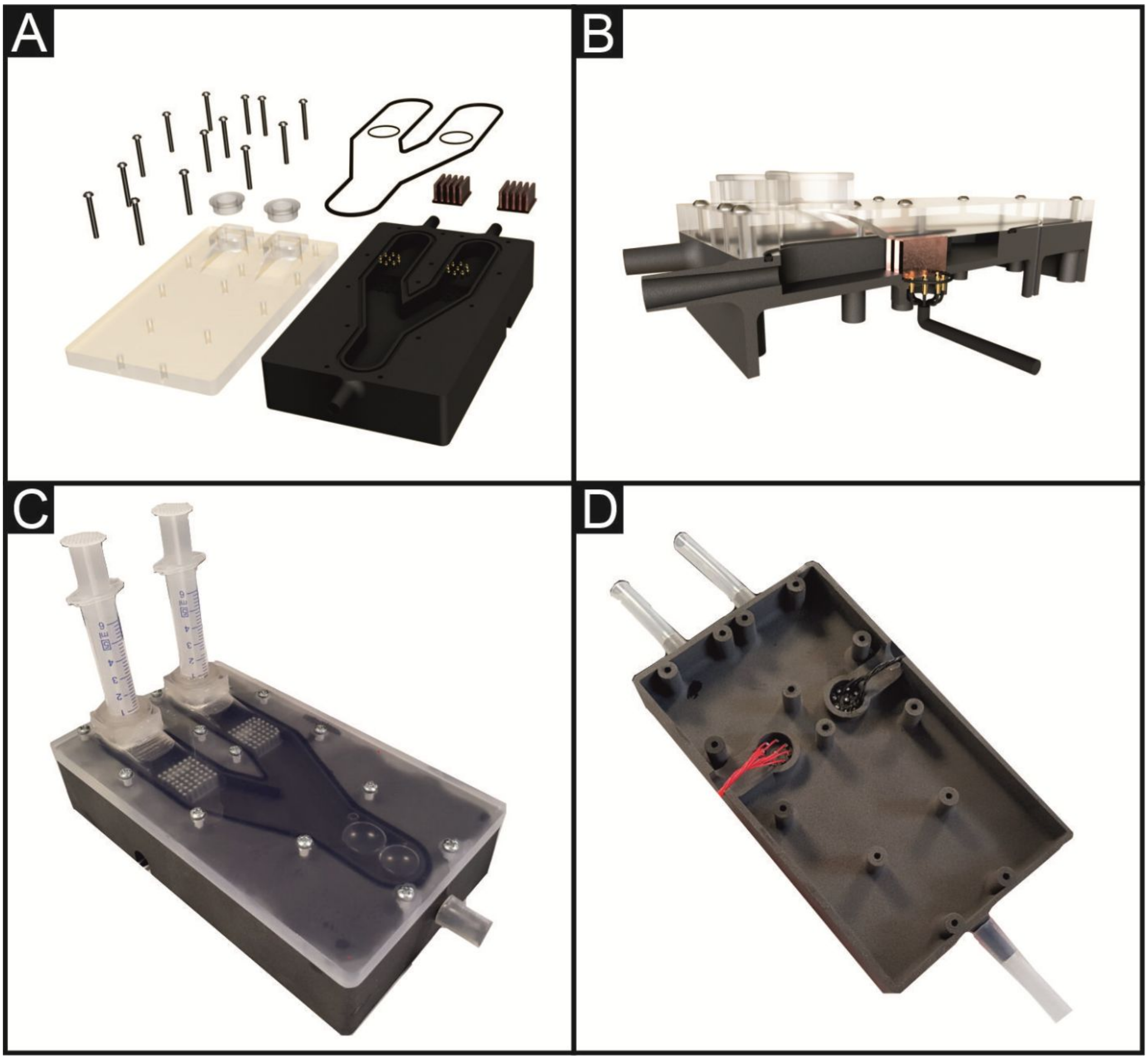

References:

1 O'Neil, G. D., Christian, C. D., Brown, D. E. & Esposito, D. V. Hydrogen Production with a Simple and Scalable Membraneless Electrolyzer. *J. Electrochem. Soc.* **163**, F3012-F3019 (2016). https://doi.org:10.1149/2.0021611jes
